# Converging paths for microscale energy storage and sensing

**DOI:** 10.1093/nsr/nwaf511

**Published:** 2025-11-17

**Authors:** Minshen Zhu, Oliver G Schmidt

**Affiliations:** Research Center for Materials, Architectures and Integration of Nanomembranes (MAIN), Chemnitz University of Technology, Germany; Material Systems for Nanoelectronics, Chemnitz University of Technology, Germany; Research Center for Materials, Architectures and Integration of Nanomembranes (MAIN), Chemnitz University of Technology, Germany; Material Systems for Nanoelectronics, Chemnitz University of Technology, Germany; International Institute for Intelligent Nanorobots and Nanosystems (IIINN), Fudan University, China

The vision of wearable microsystems that can continuously monitor physiological and biochemical signals has long inspired the healthcare electronics community [1]. Such devices could transform health management by enabling real-time diagnostics and personalized rehabilitation. However, their practical deployment remains constrained by a simple but profound problem: how to build systems that are energy-autonomous and seamlessly integrated. The bottleneck is not a lack of functional components but the challenge of integrating energy storage, power delivery and sensing modules without compromising compatibility, robustness or manufacturability.

**Figure 1.**
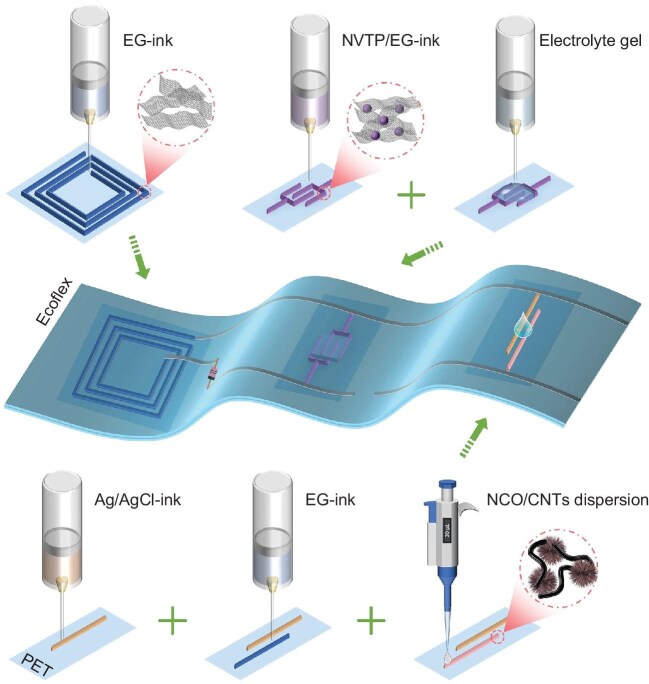
Schematic illustration of the fabrication process for the stretchable integrated microsystem. EG: exfoliated graphene; NCO: NiCo_2_O_4_; CNT: carbon nanotube. Reproduced with permission from ref. [2].

In a recent study published in *National Science Review*, Ren and colleagues present a notable step: a fully 3D-printed modular microsystem that integrates wireless charging coils, aqueous sodium-ion micro-batteries and glucose sensors into a single stretchable platform [2]. Their strategy relies on a binder-free graphene ink, formulated with glycerol, which serves as both a wireless coil and a conductive substrate for biosensing (Fig. 1), thereby simplifying the construction process. For energy storage, they employ a Na_2_VTi(PO_4_)_3_ (NVTP)-based composite ink to print a sodium-ion battery, paired with a ‘water-in-salt’ 30 m (mol/kg) sodium trifluoroacetate electrolyte. This combination delivers a wide electrochemical window of 3.2 V, allowing stable operation across multiple redox plateaus. The printed battery achieves an areal capacity of 0.96 mAh cm^−2^, an energy density of 1.24 mWh cm^−2^ and long-term stability over 1500 cycles at high rates, with performance maintained, even at sub-zero temperatures.

Equally compelling is the systems-level design. By varying printing layers, Ren and colleagues tune output capacity and voltage in serial or parallel configurations. Liquid-metal interconnects and Ecoflex elastomer encapsulation form an ‘island–bridge’ architecture that decouples mechanical strain from electrochemical function. The devices withstand 50% stretching and repeated bending, folding and twisting, retaining nearly full electrochemical capacity after 1000 deformation cycles. At the microsystem level, the wireless coils reliably charge the printed battery, which in turn powers an NiCo_2_O_4_ glucose sensor with sub-second response time and sensitivity down to 0.1 mM. The entire integrated system continues to operate stably under repeated mechanical stress, highlighting its readiness for real-world wearable use.

What distinguishes this work is not just its performance metrics, but its integration. It demonstrates that with the right materials palette and structural design, microscale energy storage need not be a limiting afterthought but an intrinsic enabler of system-level function. This work addresses the broader challenge in microscale energy research [3]; powering tiny robots, sensors and ‘smart dust’ requires not only better materials but also radically rethought architectures that shrink, fold and integrate energy devices directly into electronic systems. It also resonates with emerging design principles that emphasize that microscale energy storage is shifting from being a peripheral challenge to a central design principle that defines what microsystems can achieve in practice.

Such convergence could mark a turning point for both wearable healthcare and environmental electronics. The field has long chased incremental gains in battery capacity or sensor sensitivity [4]; this work reminds us that the more radical leap comes from rethinking integration itself. As modular, stretchable microsystems mature, they may evolve from laboratory curiosities into deployable tools for continuous health monitoring, soil sensing and beyond.
